# Supplementary material for: Malaria prevalence, knowledge and associated factors among household heads in Maygaba town, Ethiopia
Source: PLOS Glob Public Health. 2022 Mar 22;2(3):e0000071. doi: 10.1371/journal.pgph.0000071 (PMC10022035; doi:10.1371/journal.pgph.0000071)
Supplement: S1 Text — (DOC) [file pgph.0000071.s001.doc]

# Supplementary Information (S1)

# Questionnaire (መጠይቕ)

# English and Tigrigna versions

**Part I. Socio-demographic characteristics of households**

**1ይ ክፋል፡ ናይ ተሳታፊ ግላዊ ሓበሬታ**

1. Sex: a) Male b) Female

ፆታ፡ ሀ) ተባዕታይ ለ) ኣንስታይ

1. Age: _____________

ዕድመ፡------------------

1. Family size: ________________

በዝሒ ቤተ-ሰብ-------------------

1. Level of education. a) Illiterate b) Elementary school c) Secondary school and above

ትምህርት ደረጃ፡ ሀ) ዘይተምሃረ ለ) መባእታዊ ቤት ትምህርቲ ሐ) ካልኣይ ደረጃ ቤት ትምህርትን ልዕሊዑን

1. What is your livelihood? a) Farmer b) Marchant c) Student d) House wife e) Daily laborer

መተሓዳደሪኻ/ኺ እንታይ እዩ? ሀ) ሓረስታይ ለ) ነጋዳይ ሐ) ተመሃራይ መ) በዓልቲ-ሓዳር ሠ) መዓልታዊ ሰረሓተኛ

1. Do you have livestock? a) Yes b) No

ጥሪት ዶ አለካ/ኪ? ሀ) እወ ለ) ኣይፋል

1. In which village do you live? ___________________

አበይ ከባቢ ትነብር/ሪ?---------------------------

1. What kind of house do you have?: a) Conventional house b) Modern house

ብኸመይ ዓይነት ዝተሰረሐ ገዛ አለካ/ኪ? ሀ) ብባህላዊ ለ) ብዘመናዊ

**Part II. Knowledge about the cause, transmission, prevention and control of malaria**

**2ይ ክፋል፡ ግንዛበ ብዛዕባ ዓሶ ጠንቂ፣ዝመሓላለፈሉ መንገዲ፣መከላኸሊኡን መቆፃፀሪኡን**

1. Have you ever heard of malaria? a) Yes b) No

ዓሶ እንታይ ምኳኑ ቅድሚ ሕይ ሰሚዒኻ/ኺ ትፈልጥ/ጢ ዶ? ሀ) እወ ለ) ኣይፋል

1. Is malaria a communicable disease? a) Yes b) No c) I don’t know

ዓሶ ተመሓላላፊ ሕማም ድዩ? ሀ) እወ ለ) ኣይፋል ሐ) ኣይፈለጥኩን

1. What is the cause of malaria? a) Mosquito bite b) Environmental change c) Lack of environmental sanitation

ጠንቂ ናይ ዓሶ መምፅኢ እንታይ እዩ? ሀ) ንክሲት ጣንጡ ለ) ከባቢያዊ ለውጢ ሐ) ከባቢያዊ ፅሬት ብዘይ ምሕላው

1. When do mosquitos bite mostly? a) In the day b) In the night c) Anytime

ብዝበለፀ ጣንጡ ትናኸሰሉ ጊዜ መዓዝ እዩ? ሀ) ቀትሪ ለ) ለይቲ ሐ) አብ ዝኾነ ጊዜ

1. Is malaria preventable? a) Yes b) No c) I don’t know

ሕማም ዓሶ ምክልኻል ይካኣል ዶ? ሀ) እወ ለ) ኣይፋል ሐ) አይፈለጥኩን

1. What are the prevention methods? a) Apply environmental sanitation b) Use of ITN c) Fumigation d) Wearing long sleeved clothes

ናይ ዓሶ መከላኸሊ መንገድታት እንታይ እዮም? ሀ) ከባቢያዊ ፅሬት ምሕላው ለ) ኬሚካል ዝተዓለኸ ዛንዜራ ምጥቃም ሐ) ብትኪ ምዕጣን መ) ሙሉእ አካላትካ ዝሽፍን ክዳን ምኽዳን

1. Is malaria treatable? a) Yes b) No c) I don’t know

ዓሶ ክሕከም ዝኽእል ሕማም ድዩ? ሀ) እወ ለ) ኣይፋል ሐ) አይፈለጥኩን

1. If your answer for question number 15 is yes, what are your ways of treatment? a) Traditional healer b) Health center c) Buy drug from pharmacy d) Others_____________

እንድሕር ንሕቶ ቁፅሪ 15 እወ ኮይኑ መልስኻ/ኺ፤ ኣየና ናይ ሕክምና ሜላ ትጥቀም/ሚ?

ሀ) ባህላዊ ሕክምና ለ) ጥዕና ማእኸል ሐ) ካብ ቤት መድሓኒት መድሓኒት ምዕዳግ መ) ካሊእ መማረፂ እንተሃልዩ ግለፅ/ፂ----------------------

**Part III. Participant’s knowledge, ownership and use of ITNs**

**3ይ ክፋል፡ ተሳተፍቲ ብዛዕባ ዛነዜራ ኣጠቓቕማ ዘለዎም ግንዛበን ዋንነትን**

1. Have you ever heard about ITN? a)Yes b) No

ዛንዜራ እንታይ ምኳኑ ሰሚዒኻ/ኺ ዶ ትፈልጥ/ጢ? ሀ) እወ ለ) ኣይፋል

1. If your answer for question 17 is yes, what are the sources of information? a) Mass media (TV & radio) b) Health workers c) Local leader

እንድሕር ንሕቶ ቁፅሪ 17 እወ ኮይኑ መልስኻ/ኺ፤ ፍልፍል ሓበሬታኻ/ኺ እንታይ እዩ?

ሀ) መራኸቢ ሓፋሽ (ቴሌቪዥንን ራዲዮን) ለ) ሰራሕተኛታት ጥዕና ሐ) አመሓደርቲ

1. Does your household have any ITNs? a) Yes b) No

ስድራ ቤትካ/ኪ ዛንዜራ ኣለዋ ዶ? ሀ) እወ ለ) ኣይፋል

1. If your answer for question 19 is yeas, how many ITN do you have per family?________

እንድሕር ንሕቶ ቁፅሪ 19 እወ ኮይኑ መልስኻ/ኺ፤ ኣብ ነፍሲ ወከፍ ሕድሕድ ሲድራ ቤት ክንደይ ዛንዜራ ኣሎ?____________________

1. If your answer for question 19 is no, what are the reason for not having ITN? a) Worn-out b) Not received

እንድሕር ንሕቶ ቁፅሪ 19 ኣይፋል ኮይኑ መልስኻ/ኺ፤ ስድራ ቤትካ/ኪ ዛንዜራ ንኸይህልዋ ምኽንያት ዝኾነ እንታይ እዩ? ሀ) አሪጉ ተሰንጉዩ ለ) አይተዋህብናን

1. When did you receive the ITNs? a) More than two years b) One to two years c) Less than one year.

ዛንዜራ ካብ ዝዕደለካ/ኪ ኪነደይ ጊዜ ኮይኑካ/ኪ? ሀ) ልዕሊ 2 ዓመት ለ) ካብ 1-2 ዓመት ሐ) ትሕቲ 1 ዓመት

1. Is sleeping under ITN beneficial for the family members? a) Yes b) No

ኣብ ውሽጢ ዛንዜራ ምድቃስ ንስድራ ቤት አባላት ረብሓ ኣለው ዶ? ሀ) እወ ለ) ኣይፋል

1. If your answer for question 23 is yes, what are the benefits of ITN? a) Protect from mosquito bite b) Comfortable for sleep c) Others please specify_____________________

እንድሕር ንሕቶ ቁፅሪ 23 እወ ኮይኑ መልስኻ/ኺ፤ እቶም ናይ ዛንዜራ ረብሓታት እንታይ እዮም?

ሀ) ካብ ጣንጡ ንኪሲት ይከላኸል ለ) ንምድቃስ ምችው እዩ ሐ) ካሊዕ ረብሓ ዕንተሃልዩ ግለፅ/ፂ-______________________________

1. Did anyone of your family member sleep under ITNs last night? a) Yes b) No

ትማሊ ምሸት ኣብ ዛንዜራ ውሽጢ ዝደቀሰ ኣባል ስድራ ቤትካ/ኪ ኣሎ ዶ? ሀ) እወ ለ) ኣይፋል

1. How often do you use ITN? a) Regularly b) During peak malaria seasons c) Sometimes

ንክንደይ ሻዕ ዝኣክል ዛንዜራ ትጥቀም/ሚ? ሀ) ኩሉሻዕ ለ) ሕማም ዓሶ ኣብ ዝተስፋሕፍሓሉ ወቕቲ ሐ) ሓደሓደ ጊዜ

1. Who among the family members commonly use ITNs? a) Pregnant women b) Mother and children

ኣብ ውሽጢ ኣባላት ስድራ ቤትካ/ኪ ዛንዜራ ኣዘውቲሩ ዝጥቀም መን እዩ? ሀ) ጥኑሳት ኣንስቲ ለ) ኣደን ቆልዑን

1. Since you got the ITNs, was it ever re-treated with chemical/insecticide? a) Yes b) No

ዛንዜራ ተቀቢልካ/ኪ ምስተጠቀምካሉ/ክሉ ተመሊሱ ፀረ-ተሃዋስ ኬሚካል ይእለኽ ዶ? ሀ) እወ ለ) ኣይፋል

1. If your answer for question 28 is no, what are the reasons? a) Lack of awareness b) Lack of chemicals/insecticides

እንድሕር ንሕቶ ቁፅሪ 28 ኣይፋል ኮይኑ መልስኻ/ኺ፤ ምኽንያቱ እንታይ እዩ? ሀ) ናይ ግንዛበ ጉድለት ለ) ናይ ኬሚካል ሕፅረት
