# Supplementary material for: Malaria prevalence, knowledge and associated factors among household heads in Maygaba town, Ethiopia
Source: PLOS Glob Public Health. 2022 Mar 22;2(3):e0000071. doi: 10.1371/journal.pgph.0000071 (PMC10022035; doi:10.1371/journal.pgph.0000071)
Supplement: Attachment — Submitted filename: Response to reviewers.doc [file pgph.0000071.s002.doc]

28 August 2021

Dear, David Musoke, PhD

Academic Editor

PLOS Global Public Health

We are very much thankful to the academic editor and the reviewer (s) for detailed, prized, treasurable and comprehensive comments to the revised version of our manuscript (PGPH-D-21-00037). In the revised version of MS, all the issues raised by the reviewer (s) such as sampling techniques, data analysis, methods, discussion and results sections were properly corrected and edited. We have also thoroughly corrected the English issues of the manuscript and significantly improved the manuscript content accordingly. We hope you will now find the manuscript suitable for publication in the PLOS Global Public Health.

Yours sincerely,

The following are point-by-points response to the reviewer’s comments.

Reviewer #1:

Title

The title of the study is long and the key outcome of interest is not clear. The study population of interest is not mentioned in the title. The specific study area should be mentioned as well. The study title could be rephrased to read “Malaria prevalence, insecticide-treated mosquito net use and associated factors among household heads in Maygaba town, Ethiopia”

***Response****: We thank the reviewer for valuable points and corrected according to the comment*

**Abstract**

Abstract

The introduction should highlight key issues on malaria prevalence, use of ITNs and associated factors. The study aim should align with the suggested study title. Include information on study population, sampling, and key outcome measures in the study methodology. There should be results for key study objectives that should be aligned with the proposed study title. When describing associations, show how it influences the outcome of interest. The conclusion and recommendation should be rewritten to focus on the key outcomes of interest. It is not true that malaria prevalence was high (4.7%) in the study population.

***Response:*** *We would like to thank the reviewer for valuable points and corrected according to the comments*

**Introduction**

The introduction is very long and not well focused on the title of the study. The authors have largely dwelt on the global and local malaria burden and control measures. It would be helpful to outline why the problem or question is important. Focus on what is known about malaria burden and use of insecticide-treated mosquito net for malaria control and associated factors among household heads. Recent literature on what is known about prevalence and factors associated with use of insecticide-treated mosquito nets should be included in the introduction. The authors should state the relevance of the research to other research that has been done. Please include contextual information on what is being done to control malaria in the study area.

***Response****: Thank you very much for the critical comments and suggestions, all the issue were properly corrected in the revised MS.*

**Materials and Methods:**

**Study design, area and population.**

The study design and area is well described. The authors should give a strong justification for carrying out the malaria prevalence study among adults and the utility of such information. Malaria burden among adults is not a true reflection of malaria burden in the community because adults have partial immunity to malaria in endemic areas. Malaria prevalence studies are routinely done in children because they are not immune to malaria and carry the highest burden of malaria.

***Response****: The reviewer has raised valuable issues, but the main reason that the study site is located in the boarder of two different regions of Ethiopia with many social, economic and political conflicts. Particularly, the original people were displaced, marginalized and indirectly attacked by vaccine to make the people infertile for the sack of clearing from the area. Due to such complicated political reasons people are suspicious and not willing to donate bloods for any reasons including research and taking such issues as a tradition taboo. For this reason, we only convince the adults (>18 year old) community members for the parasitological study. But we cannot put this reasons in the manuscript since such issues are offensive to others.*

Please justify why information on net use was collected for one person in a household. Information on use of bednets is best collected from all household members in a selected house hold as per the Roll Back Malaria (RBM) indicators.

***Response****: Thanks the reviewer for the valued question. There are many reasons that we couldn’t survey the whole household members following the RBM indicators. The first reason is that the survey was conducted during the small rainy season, thus due to intense farming activity, we cannot obtain the whole family at home, second, the time was also a critical time for students to take exam and students are travelled distance to go to school again we cannot obtain the whole family at once during survey. Third the area is politically unstable as mentioned somewhere in this response letter, thus this was significantly hindering the data collection procedure including RBM protocol.*

The population inclusion and exclusion criteria should be well stated including consent to participate in the study.

***Response****: The inclusion and exclusion criteria were stated in the method part and the consent to participation were indicated in the ethical statement section.*

**Sample size and sampling**

It is not clear what key outcome measure was used for sample size estimation. The authors should use one key outcome to estimate sample size. The value of “p” was assumed as 0.5 yet there is existing literature from which values can be got, please clarify why a value from literature was not used.

***Response****: The reviewer has raised valued points, initially we thought there is no research findings exactly in the study site, later after designing and sample size determination, we learnt that “p” value could be taken from literatures published in the nearby of the study site. We greatly appreciate the comments.*

The authors state that they used a multi-stage cluster random sampling technique. To adjust for the variations between the clusters, the authors should have adjusted sample size by a design effect and possibly used a different formula for sample size estimation, please clarify why this was not the case.

***Response****: We also greatly appreciated the reviewer for the valued point raised. The reason fo not used different formula for each cluster was as we mentioned in the method section from lines 114-124, nearly all the clusters in multistage stage had the same proportion except villages with little variation. Thus, we thought as the same proportion were appropriate for the sample size determination. However, we still regret that if we used the different formula may help to avoid such issues.*

The authors state that 3 villages (Korarit, Maygaba and Adijamus) with a total population of 16,781 and 3,814 households were sampled out of the 6 villages in the study area. Please state the criteria for selecting only 3 villages and how sampling was done for the 3 out of the 6 villages in the study area.

***Response****: As it indicated in the MS with Fig 2, all the steps were implemented a* *multi-stage cluster random sampling technique. We don’t use any specific criteria for selection except random sampling technique. Since all six villages have equally chance to get selected and have same malaria problem.*

Another confusing tier for sampling is the “Gujiles”. The authors should clearly distinguish between a Gujile and a village and how they interrelate. Clearly outline how households were selected from the “villages/Gujiles”.

***Response****: As we have tried to elaborate clearly on flow chart in Fig. 2, the village is the higher social cluster, and each villages contains about 28 Gujlies on average. On the other hand each Gujile also contains about 45 households on average. This is clearly indicated in the revised MS.*

Systematic random sampling requires a list of households which has not been mentioned by the authors. The ideal sampling criterion should have been sampling proportionate to size. The authors should further describe how the respondents were sampled from each household.

***Response****: It is known that systematic random sampling was conducted after getting list of households, thus, in this study we got a list of each household in each Gujile prior to sampling. As we indicated in the MS, “This procedure was used because Gujiles are constructed from approximately equal numbers of households.” So sampling was proportionate to size. However, writing everything in the MS is cumbersome for readers.*

**Data collection and analysis**

The authors should describe the key outcome measures and for each give a detailed description of how the outcome was measured. The authors have coined a composite index they refer to as “knowledge about malaria and ITN utilization”. Any composite index used should be a meaningful index.

***Response****: We also acknowledged the points raised here, the authors were scored the knowledge levels as indicated in the “scoring system”. However, we don’t use any composite index such as “Knowledge about malaria and ITN utilization”. However, by mistake the tem were appeared on the MS during writ up. We grateful acknowledged the issue and corrected throughout the revised MS.*

The authors state that questionnaires were pre-tested by preliminary survey in some *Gujiles*, please clarify if these were outside the sampled areas.

***Response****: We thank the reviewer for the issue, corrected in the revised MS*

Describe in detail how quality control of smear reading was done and how discrepant readings were resolved.

***Response****: We thank the reviewer for valuable comments, corrected as it suggested.*

In the data analysis please describe how you addressed confounding.

***Response****: The issue was addressed as it suggested.*

**Results:**

The results section should be re written. Results should be presented per objective. Results for key outcomes such as parasite prevalence, awareness about cause, transmission, control and prevention of malaria, knowledge about malaria, and use of ITNs should initially be presented separately. A bivariate analysis of the dependent and independent variables can then be made thereafter.

***Response****: We thank the reviewer for valuable comments, corrected as it suggested.*

To adjust for confounding, a multivariable analysis should be done possibly using odds ratios or prevalence ratios for the analysis.

***Response****: We thank the reviewer for valuable comments, following the comments we have done logistic regression as you suggested and presented it with Table 6.*

The authors should use the recommended RBM indicators to describe net use. Please note that history of fever or temperature measurement were not taken on study participants so you in effect did not determine prevalence of asymptomatic malaria parasitaemia.

***Response****: We thank the reviewer for valuable comments, since the data has already generated and cannot undo, authors are respectfully acknowledge the issue and will take it as good lessons for future research.*

**Discussion:**

The discussion is long but not focused on study objectives. The discussion should focus primarily on results from all the study objectives. The authors should discuss the significance of all key findings before relating to other researched work.

***Response****: Thank you very much for the valuable points and corrected as you suggested*

The authors should discuss if results from the study can be generalized to other areas of the same setting. Study limitations should be comprehensively discussed including the limitations in the study design.

***Response****: Authors are accepted the comments and corrected as you suggested*

Comment on whether results can be generalized to other settings in Africa and across the world. There should be a conclusion for every objective and recommendations should be based on the study finding.

***Response****: Authors are accepted the comments and corrected as you suggested*

Reviewer #2:

Comments:

All comments and suggestions uploaded; I think, in this study, what was actually measured was positivity rate but not prevalence. I would strongly recommend a change. Otherwise, if it is a prevalence, let the try to do weighted analysis.

***Response****: We really appreciated the reviewer for the valuable points raised. We had a correction on the title following the suggestion obtained from the first reviewer. Plus to this, the prevalence study was focused only on the household heads (> 18 years old) as we mentioned the main reasons in the methodology section of the MS. Authors are also respectfully acknowledge the issue and will take it as good lessons for future research. We believe that the above reasons may convince you to accept our MS for consideration.*

Again, in the title, the term “practices: and use more or less mean the same. May think about dropping use and remain with the KAP.

***Response****: Thank you very much for the comment, corrected as you suggested*
