# Supplementary material for: Malaria prevalence, knowledge and associated factors among household heads in Maygaba town, Ethiopia
Source: PLOS Glob Public Health. 2022 Mar 22;2(3):e0000071. doi: 10.1371/journal.pgph.0000071 (PMC10022035; doi:10.1371/journal.pgph.0000071)
Supplement: Attachment — Submitted filename: Response to reviewers.doc [file pgph.0000071.s003.doc]

16 October 2021

Dear, David Musoke, PhD

Academic Editor

PLOS Global Public Health

Once again thank you so much the academic editor and the reviewer (s) for detailed, prized, treasurable and comprehensive comments to the revised version of our manuscript (PGPH-D-21-00037R1). In the second revised version of our MS, all the issues raised from the academic editor, reviewer (s) and journal editorial office were properly corrected and edited. By now, the revised manuscript is significantly improved. Hopefully, the MS is suitable for publication in the **PLOS Global Public Health**.

Yours sincerely,

The following are point-by-points response to the academic editor and reviewer comments.

Additional Editor Comments:

***Q#***. May you revise the manuscript based on the latest comments, paying particularly attention to thorough English editing.

***Response***: Thank you very much for the comment raised by the academic editor. We have thoroughly corrected the English issues of the manuscript and significantly improved the manuscript content accordingly. The corrected points were indicated in the marked version with track change.

Reviewer #1:

***Q#1***. **Abstract section**

- The statement “Reports indicated that consistent and proper utilization of ITNs reduce transmission rate of malaria by 90%” should be rephrased to read “Consistent use of ITNs reduces malaria transmission.
- The statement “However, educational level, knowledge of malaria, socio-economic status and parity and community participation were the major factors for the prevalence of malaria in the rural settings” is not clear. A clear statement on malaria burden and associated factors should be made.
- The statement “The study was aimed to assess” should be rephrased to read “ We assessed”.
- Specify that blood samples were collected from household heads or their designate.
- Details about the statistical package for analysis could be deleted from the abstract.
- The statement “Generally 403 blood samples were examined, of which 19 (4.7%) were confirmed to be malaria parasite carriers (12 cases of P. falciparum and 7 cases of P. vivax )” should be rephrased to read as “Of the 403 blood samples, 19 (4.7%) had malaria parasites (12 cases of P. falciparum and 7 cases of P. vivax )”
- The statement “Among 64% (257) of the respondents that owned ITNs, only 66.9% (172) of them consistently used the ITNs during the peak season” should be rephrased to read “About 64% (257) of the respondents owned ITNs, of these, 66.9% (172) consistently slept under the nets during the peak season.
- The authors state that Chi-square tests showed a significant ( p = 0.002) association between respondents’ knowledge about malaria and ITNs utilization”. Its not clear if knowledge was associated with increased or reduced use of nets, this should be clear in the analysis and reporting.
- The statement “ Logistic regression analysis revealed that adults whose age group is 18-30 were significantly (AOR = 5.982; 95% CI = 1.715- 20.863; p-value = 0.002) more affected by malaria than any other age group” should be rephrased to read “Malaria infection was significantly higher among household heads aged 18-30 years (AOR = 5.982; 95% CI = 1.715- 20.863)”.
- As stated earlier, the conclusion and recommendation should be rewritten to focus on the key outcomes of interest (malaria infection prevalence, use of ITNS and associated factors).

***Response***: Thank you so much for the valuable comments raised by the reviewer in the abstract section. Each and every comment raised in the abstract section was thoroughly corrected and amended to the revised MS version. The corrections of each and every point raised were indicated by track change in the unmarked version of the manuscript.

***Q#2***. **Introduction section**

- The first time you use an abbreviation, write it in full.
- Line 58 is not clear, clarify if you meant “the least (20.7%) was reported in the lowlands [11]”.
- Delete the statement “Hence, studying the knowledge, attitudes and practices (KAP) towards the use of ITNs in such limited resources is vital beside other efforts made to control malaria in the region. Furthermore, understanding the current status of malaria prevalence as well as integrated KAP towards the utilization of ITNs, completes the protection and control efforts of malaria in the region, especially in the study area”
- ***Response***: Thank you so much again for the valuable comments in the introduction section. Corrected as you suggested and indicated by track change in the unmarked version of the manuscript.

***Q#3***. **Materials and methods (Study design, area and population).**

- Line 92 should read “The population size of each selected village…”.
- Line 93 should read “On average, each village had 1,271 households and 5,593 persons…”
- Clarify what you meant by “masl” in line 95.
- Line 97 should read “Persons eligible for the parasitological survey were heads of households or their designate willing to give blood samples for malaria testing and with no history of ..”
- Delete the statement the following statement from line 99 to 102 to avoid confisong readers: “Any family members who were unable to communicate, mentally retarded, and severely ill were excluded from the study. However, due to traditional taboos of the community, parents were not willing to let their children (< 18 years) participate in the study as blood smear donors.
- Delete the statement “Actually 403 participants (95.5% of response rate) were completed the KAP and parasitological studies” from line 113 since this section is describing methods.
- In line 115 delete the word “cluster” since this is not a randomised cluster trial. Note that randomised stratified sampling was used.

***Response***: We thank the reviewer for critical comments and observations in the method section (Study design, area and population). All the suggestions that need to delete, were deleted, and need to be rephrased, were rephrased following your valuable comments and indicated by track change in the unmarked version of the manuscript.

***Q#4***. **Materials and methods (Data collection and analysis).**

- The authors should describe the key outcome measures and for each give a detailed description of how the outcome was measured. The authors are using a composite index they refer to as “overall KAP”. Please clarify what this outcome means.
- Describe how discrepant smear readings were resolved.
- Clarify what you meant by the statement “the study design was randomized to reduce potential confounding in line 157.
- You also used odds ratios in data analysis, this should be mentioned in the text.

***Response***: We thank the reviewer for critical comments in the method section (Data collection and analysis). The key outcome measures were described in detail, the discrepant smear reading resolution was added to the section and other comments were amended according to the comments. The corrections were indicated by track change in the unmarked version of the manuscript.

***Q#5***. **Results.**

- Line 178 should read “Majority of the households had greater than five….”.
- Line 186 should read “Of the 403 respondents, 240 (61.5%) were male….”
- Delete the sentence following sentence fond in lines 87 to 90 “A total of nineteen slide-positives were obtained, of which twelve cases were obtained from male and seven cases were from female participants. Of the nineteen asymptomatic malaria cases, sixteen cases of malaria were obtained from the age group of years old blood film donors.
- Line 200 should read “A total of 239 respondents….”

***Response***: We thank the reviewer for valuable comments in this section. All the issues were taken and corrected. The corrections were indicated by track change in the unmarked version of the manuscript.

***Q#6. Continued from result section***

- Malaria knowledge is not a key outcome measure therefore it is not necessary to show the association of malaria knowledge with socio-demographic data .Your key outcome measures are malaria infection and net use. Include results of a bivariate analysis of these key outcome measures (like table 6 for malaria infection) and the associated factors and thereafter do a multivariable analysis to adjust for confounding factors

***Response***: Thank you very much for the critical and very important comment which significantly improved the manuscript. This manuscript is prepared from an M.Sc thesis conducted under our supervision at Addis Ababa University; the student who collected the data is the first author in the manuscript. All the row data is with the student. Due to the current escalated regional conflict we are unable to reach the M.Sc student, because of which it may be difficult for us to do the relationship between the socio-demographic factors and one of our outcome variable (ITNs) utilization.

***Q#7. Discussion section***

- Update the discussion with a discussion of factors associated with net use once the results are presented.

***Response***: Thank you so much for the valuable comments in this section, we updated the discussion by adding proper phrases from reference literature. You may see it in the correction in the track change version of the manuscript.

***Q#8. General comments***

- The paper needs further minor revisions.
- The paper may benefit from a language edit.

***Response***: We gratefully thank the reviewer for all the effort made to bring the fundamental modification on our manuscript. This manuscript was not only edited on minor revision alone but also carefully edited the English issues raised by both academic editor and reviewer (s) of the manuscript. Finally, we would like to thank the academic editor, reviewers and journal editorial team members for your time, endurance, and kindness to our manuscript.

**May God Bless you all**!!!!!

Other comments from Journal office:

***Q#***. Please review your reference list to ensure that it is complete and correct. If you have cited papers that have been retracted, please include the rationale for doing so in the manuscript text, or remove these references and replace them with relevant current references. Any changes to the reference list should be mentioned in the rebuttal letter that accompanies your revised manuscript. If you need to cite a retracted article, indicate the article’s retracted status in the References list and also include a citation and full reference for the retraction notice.

***Response***: Thank you very much for the comments, we greatly apologize for the mistake happened to previous version of our manuscript in the reference section. Now, all the reference lists are properly checked and corrected. However, ten references listed below were retracted out from the manuscript during revision following the reviewers’ comments in the first revision (PGPH-D-21-00037), and one reference was added to the manuscript again during revision of the manuscript.

\# References retracted from both versions (PGPH-D-21-00037 and PGPH-D-21-00037R1)

1. Girum T, Shumbej T, Shewangizaw M. Burden of malaria in Ethiopia, 2000-2016: findings from the Global Health Estimates 2016. Trop Dis Travel Med Vaccines. 2019 Jul 12;5:11.
2. World health organization. World malaria report 2019. Geneva: WHO; 2019.
3. Chibsa S. Malaria Vector Control Efforts and Challenges in Ethiopia. In 4th WIN Meeting; 2007 Oct 24-26; Basel, Switzerland, 2007. p. 1–35.
4. Bugssa G, Tedla K. Feasibility of Malaria Elimination in Ethiopia. Ethiop J Health Sci. 2020 July 1;30(4):607-614.
5. World health organiztion. Indoor residual spraying: an operational manual for indoor residual spraying (IRS) for malaria transmission control and elimination. Geneva: WHO; 2015.
6. Correa APSA, Galardo AKR, Lima LA, Camara DCP, Muller JN, Barroso JFS, et al. Efficacy of insecticides used in indoor residual spraying for malaria control: an experimental trial on various surfaces in a “test house”. Malar J. 2019 Oct;18(1):345.
7. Tilaye T, Deressa W. Prevalence of urban malaria and assocated factors in Gondar Town, Northwest Ethiopia. Ethiop Med J. 2007 Apr;45(2):151-158.
8. Imperato PJ. Malaria parasitemia in healthy Africans in north Mara, Tanzania. J Community Health. 1986;11(2): 92-97.
9. Ferede G, Worku A, Getanehet A, Ahmed A, aile T, Abdu Y et al. Prevalence of malaria from blood smears examination: a seven-year retrospective study from Metema Hospital, Northwest Ethiopia. Malar Res Treat. 2013 Nov; 2013.
10. Beavogui AH, Delamou A, Camara BS, Camara D, Kourouma K, Camara R, et al. Prevalence of malaria and factors associated with infection in children aged 6 months to 9 years in Guinea: Results from a national cross-sectional study. Parasite Epidemiol Control. 2020 June;11:e00162.

\# Refernce added to the revised manuscript:

1. Deress T, Girma M. *Plasmodium falciparum* and *Plasmodium vivax* prevalence in Ethiopia: a systematic review and meta-analysis. Malar Res Treat. 2019 Dec 28.
