# Supplementary material for: Malaria prevalence, knowledge and associated factors among household heads in Maygaba town, Ethiopia
Source: PLOS Glob Public Health. 2022 Mar 22;2(3):e0000071. doi: 10.1371/journal.pgph.0000071 (PMC10022035; doi:10.1371/journal.pgph.0000071)
Supplement: Attachment — Submitted filename: Response to reviewers.doc [file pgph.0000071.s004.doc]

18 December 2021

We gratefully acknowledged the academic editor and reviewer for their valuable comments to our manuscript. The following are point-by-point responses to the academic editor and reviewer comments for the manuscript number: **Review of manuscript PGPH-D-21-00037-R2.**

Reviewer #1:

**Q#1**: The authors should mention what the key outcome measure(s) were in the methods section and describe how composite outcomes were measured.

**Answer**: *A paragraph was included in the method section that shows how outcome measures were taken during analysis.*

**Q#2**: The section on KAP scoring system needs to be made clearer. The authors stated that a KAP scoring system was adopted from a previously published method. Briefly mention what was being assessed under this system. The authors should include results for the KAP scores in the paper, if not the whole section should be deleted.

**Answer**: *This section was deleted following the suggestion.*

**Q#3**: The authors have shown results of association of malaria knowledge with socio-demographic data. Please describe how the outcome “malaria knowledge” was measured.

**Answer**: *This question was addressed in Q#1.*

**Q#4**: Please include results for factors associated with net use and discuss these findings in the discussion

**Answer**: *The authors were replied to this question in the previous response letter (PGPH-D-21-00037-R1). Furthermore, in this revision, we have modified the title to glance at the two main outcomes, namely malaria prevalence and knowledge about malaria since we conducted only a descriptive analysis to the net use.*

**Q#5**: There are still many grammatical errors in the paper, the paper may benefit from a language edit.

**Answer**: *This time, the manuscript was reviewed by native English speaker and all the changes were indicated by track change.*
